# Skeletal muscle insulin resistance is induced by 4-hydroxy-2-hexenal, a by-product of *n*-3 fatty acid peroxidation

**DOI:** 10.1007/s00125-017-4528-4

**Published:** 2018-01-03

**Authors:** Christophe O. Soulage, Laura Sardón Puig, Laurent Soulère, Bader Zarrouki, Michel Guichardant, Michel Lagarde, Nicolas J. Pillon

**Affiliations:** 1 Univ Lyon, CarMeN, INSA-Lyon, Inserm UMR 1060, INRA UMR 1397, Université Claude Bernard Lyon 1, Villeurbanne, France; 2 Department of Molecular Medicine and Surgery, Karolinska Institutet, Stockholm, Sweden; 3 Univ Lyon, INSA-Lyon, CPE Lyon, Université de Lyon 1, UMR 5246, CNRS, ICBMS, Institut de Chimie et de Biochimie Moléculaires et Supramoléculaires, Chimie Organique et Bioorganique (COB), Villeurbanne, France; 4 Bioscience Diabetes, Cardiovascular and Metabolic Diseases, IMED Biotech Unit, AstraZeneca, Gothenburg, Sweden; 5 Department of Physiology and Pharmacology, Karolinska Institutet, von Eulers väg 4a, IV, SE-171 77 Stockholm, Sweden

**Keywords:** 4-HHE, Insulin resistance, Lipid aldehyde, Peroxidation, Skeletal muscle

## Abstract

**Aims/hypothesis:**

Oxidative stress is involved in the pathophysiology of insulin resistance and its progression towards type 2 diabetes. The peroxidation of *n*-3 polyunsaturated fatty acids produces 4-hydroxy-2-hexenal (4-HHE), a lipid aldehyde with potent electrophilic properties able to interfere with many pathophysiological processes. The aim of the present study was to investigate the role of 4-HHE in the development of insulin resistance.

**Methods:**

4-HHE concentration was measured in plasma from humans and rats by GC–MS. Insulin resistance was estimated in healthy rats after administration of 4-HHE using hyperinsulinaemic–euglycaemic clamps. In muscle cells, glucose uptake was measured using 2-deoxy-d-glucose and signalling pathways were investigated by western blotting. Intracellular glutathione was measured using a fluorimetric assay kit and boosted using 1,2-dithiole-3-thione (D3T).

**Results:**

Circulating levels of 4-HHE in type 2 diabetic humans and a rat model of diabetes (obese Zucker diabetic fatty rats), were twice those in their non-diabetic counterparts (33 vs 14 nmol/l, *p* < 0.001), and positively correlated with blood glucose levels. During hyperinsulinaemic–euglycaemic clamps in rats, acute intravenous injection of 4-HHE significantly altered whole-body insulin sensitivity and decreased glucose infusion rate (24.2 vs 9.9 mg kg^−1^ min^−1^, *p* < 0.001). In vitro, 4-HHE impaired insulin-stimulated glucose uptake and signalling (protein kinase B/Akt and IRS1) in L6 muscle cells. Insulin-induced glucose uptake was reduced from 186 to 141.9 pmol mg^−1^ min^−1^ (*p* < 0.05). 4-HHE induced carbonylation of cell proteins and reduced glutathione concentration from 6.3 to 4.5 nmol/mg protein. Increasing intracellular glutathione pools using D3T prevented 4-HHE-induced carbonyl stress and insulin resistance.

**Conclusions/interpretation:**

4-HHE is produced in type 2 diabetic humans and Zucker diabetic fatty rats and blunts insulin action in skeletal muscle. 4-HHE therefore plays a causal role in the pathophysiology of type 2 diabetes and might constitute a potential therapeutic target to taper oxidative stress-induced insulin resistance.


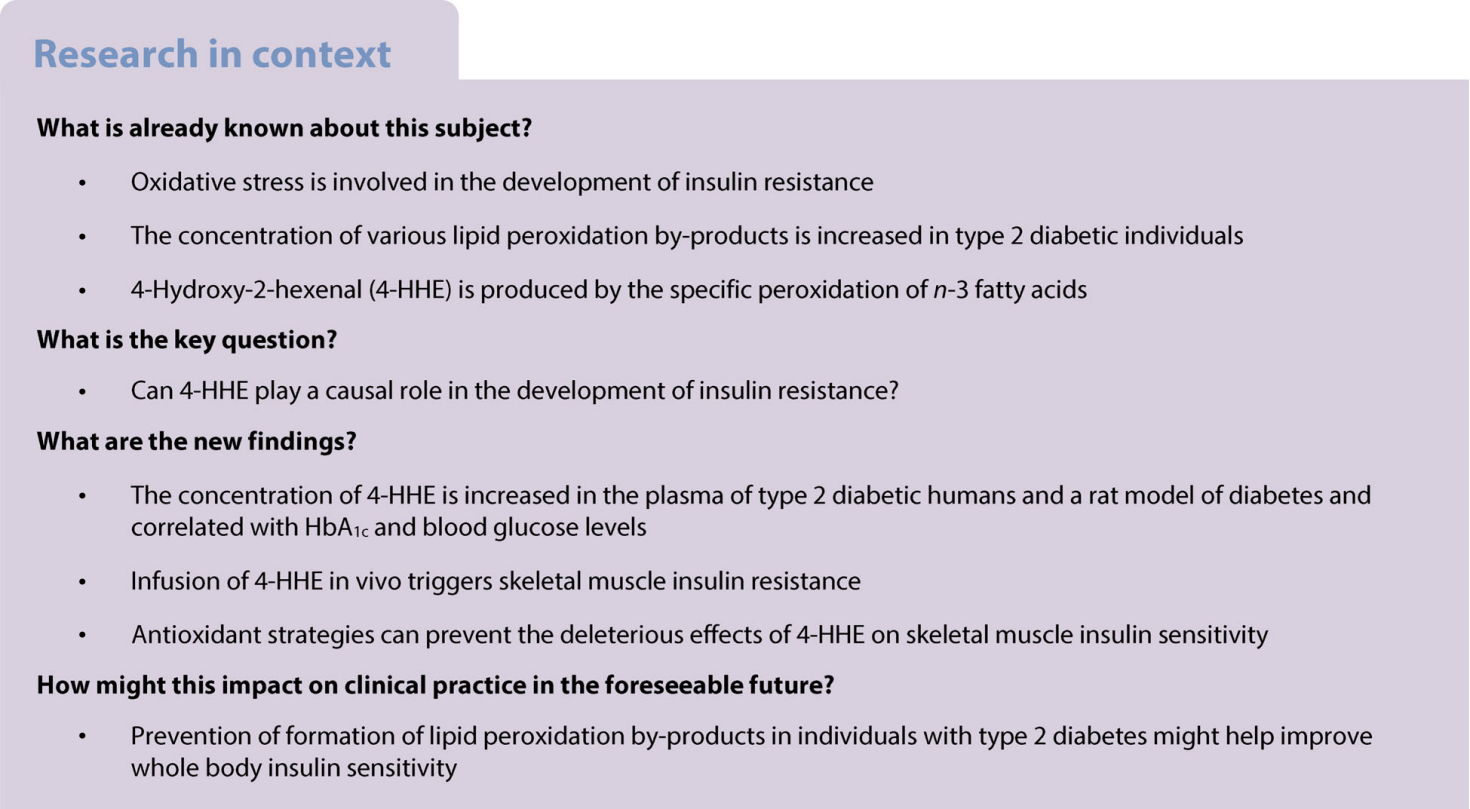


## Introduction

Oxidative stress is involved in the pathophysiology of many chronic diseases and in particular contributes to the development of insulin resistance and its progression towards type 2 diabetes [1–3]. Peroxidation of cell membrane phospholipids associated with oxidative stress produces deleterious reactive species. Peroxidation of *n*-6 polyunsaturated fatty acids (PUFA) leads to the production of 4-hydroxy-2-nonenal (4-HNE), while 4-hydroxy-2-hexenal (4-HHE) is released during the oxidation of *n*-3-PUFA [4]. These lipid aldehydes are major by-products of lipid peroxidation of PUFA and exhibit potent electrophilic properties allowing them to form covalent adducts with phospholipids, proteins and nucleotides [5, 6]. Because of their relative stability and high reactivity, these aldehydes are thought to interfere with crucial physiological processes such as cell cycle, apoptosis or metabolic pathways [7–9]. Importantly, the production of 4-hydroxyalkenals is associated with hindered insulin responses: 4-HNE-adducts accumulate in liver and pancreatic beta cells of diabetic rats [10–12], impairs glucose-induced insulin secretion in isolated beta cells [13] and blunts insulin action in 3T3-L1 adipocytes and L6 muscle cells [14, 15].

Increased consumption of *n*-3 PUFA might be expected to produce beneficial effects [16]; however, enhanced 4-HHE formation in conditions associated with oxidative stress might be harmful. For instance, consumption of oxidised *n*-3 PUFA induces oxidative stress and inflammation of mice intestine [17]. 4-HHE has, however, received little attention, despite its similarities in structure and its reactivity with related aldehydes. Indeed, 4-HHE is also produced under oxidative stress conditions, accumulates in tissues [18, 19] and is able to form adducts on biological molecules [20, 21]. Only a handful of studies have shown activation of stress signalling pathways by 4-HHE [22, 23] but data regarding its pathophysiological effects remains scarce. Especially, the putative role of 4-HHE in the development of insulin resistance has not been investigated. In the present study we hypothesised that circulating levels of 4-HHE are elevated in diabetic individuals and that 4-HHE can impair insulin responses in skeletal muscle cells and contribute to insulin resistance in vivo.

## Methods

### Reagents

4-HNE and 4-HHE were synthesised as previously described [24]. Insulin (Actrapid, 100 U/ml) was from Novo Nordisk (La Défense, France) and ECL reagent and 2-deoxy-d-2,6-[^3^H]glucose were from GE Healthcare (Orsay, France). 1,2-Dithiole-3-thione (D3T) was from Interchim (Montlucon, France). All other reagents were from Sigma-Aldrich (Saint Quentin Fallavier, France).

### Antibodies

Antibodies to Ser473-Akt, total Akt, p85 and total IRS1 (dilution 1:1000) were from Cell Signaling (Leiden, the Netherlands). Secondary antibodies (dilution 1:10,000) were from Sigma-Aldrich (Heidelberg, Germany). Anti-HHE-Michael adducts antibodies were from Cosmobio (Tokyo, Japan). Antibodies for insulin signalling were validated using insulin as a positive control. Anti-HHE antibody specificity was tested against protein lysates incubated with 4-HHE or 4-HNE.

### Participants

Fifteen individuals with type 2 diabetes mellitus and 17 healthy volunteers were recruited from an ongoing study at Hospices Civils de Lyon. The characteristics of the participants are shown in Table 1. The study was approved by the local ethics committee (reference D-09-17) and written informed consent was obtained from all volunteers. After an overnight fast, blood samples were collected, centrifuged at 1500 *g* for 10 min to isolate plasma and stored at −80°C.

**Table 1:** Demographic and clinical characteristics of participants

Characteristic	Healthy individuals	Type 2 diabetes	*p* value
Sex, *n* male/*n* female	15/2	14/1	0.738
Age, years	51 (41–57)	66 (55–74)	<0.001
BMI, kg/m^2^	23.9 ± 2.9	30.8 ± 5.0	<0.001
Fasting plasma glucose, mmol/l	5.1 (4.6–5.4)	9.4 (7.5–12.7)	<0.001
HbA_1c_, mmol/mol	34.4 (33.3–36.6)	70.5 (60.7–77.0)	<0.001
HbA_1c_, %	5.3 (5.2–5.5)	8.6 (7.7–9.2)	<0.001
Systolic blood pressure, mmHg	129 ± 13	131 ± 21	0.770
Diastolic blood pressure, mmHg	81 ± 11	72 ± 11	0.045
Total cholesterol, mmol/l	5.81 ± 1.65	3.94 ± 1.11	0.012
HDL-cholesterol, mmol/l	1.22 ± 0.31	0.88 ± 0.25	0.009
Triacylglycerols, mmol/l	1.19 ± 0.60	2.22 ± 0.87	0.003

Values are presented as mean±SD or as median (interquartile range) if data were not normally distributed

Statistical significance was determined by Student’s *t* test or Mann–Whitney *U* test. Categorical data (i.e. sex) were compared using Fisher’s exact test. *p* < 0.05 was considered significant

### Animal experiments

All animal experiments were performed under authorisation no. 69-266-0501 (INSA-Lyon, DDPP-SV, Direction Départementale de la Protection des Populations - Services Vétérinaires du Rhône) according to the guidelines laid down by the French Ministry of Agriculture (no. 2013-118) and the European Union Council Directive for the protection of animals (2010/63UE).

The rats used in all experiments were raised in an air-conditioned room with a controlled environment of 21 ± 0.5°C and 60–70% humidity, under a 12 h light–dark cycle (light on from 07:00 to 19:00 hours) with free access to food (2016C, 12.6 kJ/g; Harlan, Gannat, France) and water. Rats were housed together and randomised into groups on the day of the experiment using random numbers.

### Zucker diabetic fatty rats

Five-week-old male lean (ZDF-*Lepr*^fa^/+/?, *n* = 5) and obese Zucker diabetic fatty (ZDF) rats (ZDF-*Lepr*^fa^/Crl, *n* = 5), a rat model of diabetes, were purchased from Charles River Laboratories (L’Arbresle, France). The characteristics of the rats are shown in Table 2. At 15 weeks of age, rats were deeply anaesthetised with sodium pentobarbital (120 mg/kg i.p.). Terminal cardiac blood puncture was collected into a heparinised syringe and blood was centrifuged for 2 min at 3500 *g* to prepare plasma, snap-frozen in liquid nitrogen and stored at −80°C. Blood glucose was measured with a glucometer (Accu-Check Performa; Roche, Meylan, France) and insulin was determined using an enzyme immunoassay (EIA) according to the manufacturer’s recommendations (A05005; Bertin Pharma, Montigny le Bretonneux, France). HbA_1c_ was determined on whole blood using a rat glycated haemoglobin assay kit (80300; Crystal Chem, Zaandam, the Netherlands).

**Table 2:** Characteristics of 15-week-old lean and obese ZDF rats

Characteristic	Lean	Obese
Body weight, g	385 ± 7	729 ± 54***
Fasting blood glucose, mmol/l	4.44 ± 0.28	25.50 ± 1.33***
Fasting insulin, pmol/l	168 ± 26	427 ± 52***
HbA_1c_ (mmol/mol)	28.3 ± 1.7	73.9 ± 2.3***
HbA_1c_ (%)	4.74 ± 0.28	8.91 ± 0.28***

Values are mean±SEM, *n* = 5

***p < 0.001 vs lean (Student’s *t* test)

### Euglycaemic–hyperinsulinaemic clamps

Clamps were performed in 3-month-old anaesthetised male Wistar rats [25] purchased from Janvier (Le-Genest-Saint-Isle, France). Rats were fasted overnight, anaesthetised with sodium pentobarbital (35 mg/kg i.p.; Sanofi Santé Animale, Centravet, Lapalisse, France) and chlorpromazine (5 mg/kg, i.p., Largactil; Sanofi-Aventis, Paris, France) and then implanted with indwelling catheters (PE-20; Phymep, Paris, France) in the left carotid artery and left and right jugular veins. After catheterisation, four rats were infused with 4-HHE (10 mg/kg, 0.1 ml) and four rats were infused with the vehicle (DMSO, 0.1 ml) in the left jugular vein. A standard 2 h clamp [25] was conducted using a primed and continuous infusion of human recombinant insulin (Actrapid) at a rate of 6 mU kg^−1^ min^−1^ coupled with a variable infusion of 25% (wt/vol.) glucose to maintain blood glucose concentration at approximately 6 mmol/l. Blood glucose was measured every 5 min using a glucometer (Accu-Check Performa). Plasma insulin concentration was determined at the end of the clamp by EIA assay (SpiBio, Montigny le Bretonneux, France).

### In vivo insulin stimulation and insulin signalling in skeletal muscle

Male Wistar rats, fasted for 7 h prior to the experiments, were anaesthetised with sodium pentobarbital (35 mg/kg i.p., Sanofi Santé Animale, Centravet) and chlorpromazine (5 mg/kg i.p., Largactil; Sanofi-Aventis). Body temperature was maintained at 37°C with a homeostatic blanket (Harvard apparatus, les Ullis, France). A tracheotomy (PE-240; Phymep, Paris, France) was performed to facilitate breathing. A catheter (PE-20) was inserted into the left jugular vein for 4-HHE infusion. Rats were either infused with 4-HHE (10 mg/kg in 0.1 ml DMSO, *n* = 5) or 0.1 ml of DMSO as a control (*n* = 4). One hour after infusion, rats received an intravenous injection of either saline (154 mmol/l NaCl) or insulin (0.75 U/kg body weight). Thirty minutes after insulin injection, rats were killed with an intravenous overdose of sodium pentobarbital (120 mg/kg), blood was collected by heart puncture into a heparinised syringe and gastrocnemius muscle was rapidly dissected out, blotted dry and snap-frozen in liquid nitrogen.

### Plasma 4-HHE and 4-HNE measurement

Five hundred microlitres of plasma were used to measure 4-HHE and 4-HNE by GC–MS as previously described [26].

### Cell culture

Rat L6 muscle cells were obtained from the American Type Culture Collection (LC/GC, Molsheim, France) and grown as described previously [15]. Cells were starved of serum for 4 h before treatments, which were performed in serum-free Minimum Essential Medium Eagle Alpha Modification (Sigma-Aldrich, Saint Quentin Fallavier, France). Viability and cell death were determined by 3-(4,5-dimethylthiazol-2-yl)-2,5-diphenyltetrazolium bromide (MTT) reduction (Cell Proliferation Kit I; Roche), lactate dehydrogenase release (In vitro toxicology assay kit; Sigma-Aldrich) and caspase 3 activity (Caspase 3 Assay Kit; Sigma-Aldrich). Mycoplasma contamination was regularly tested by PCR.

### 2-Deoxy-d-[^3^H]glucose uptake

Cells were treated with 4-HHE and incubated for 20 min with 100 nmol/l insulin or 20 μmol/l cytochalasin B. Glucose uptake was initiated by the addition of 2-deoxy-d-[^3^H]glucose (747 GBq/mmol) to a final concentration of 0.1 mmol/l for 5 min at 37°C. Uptake was terminated by three washes in ice-cold PBS and solubilisation in 0.1% (wt/vol.) SDS. Tritium was detected by liquid scintillation counting and results normalised by protein concentration measured using the Bradford assay. Non-specific uptake measured in presence of cytochalasin B was subtracted from each determination.

### Spectrophotometric 2,4-dinitrophenylhydrazine assay

Carbonyl groups on proteins were determined using 2,4-dinitrophenylhydrazine (DNPH) as previously described [27]. Carbonyl content was determined from the absorbance at a wavelength of 370 nm using a molar absorption coefficient of 22,000 l mol^−1^ cm^−1^ and normalised to the protein concentration measured at 280 nm.

### Protein extraction, immunoprecipitation and immunoblotting

After incubation with 4-HHE, cells were washed with PBS and proteins were extracted as described previously [15]. For IRS1/p85 detection, whole-cell lysates were immunoprecipitated using antibodies against IRS1. Proteins were boiled in Laemmli buffer, separated by SDS-PAGE and transferred onto nitrocellulose membrane. For dot blotting, 20 μg of proteins from whole-cell lysate were loaded directly onto the nitrocellulose membrane. Following saturation with 5% (wt/vol.) BSA, membranes were probed with antibodies and processed for chemiluminescence (ECL plus; GE Healthcare, Coutaboeuf, France). Quantification was performed using the ImageJ software (National Institutes of Health, Bethesda, USA).

### Immunofluorescence

L6 cells grown on coverslips were incubated with 4-HHE, fixed for 30 min with 3% (wt/vol.) paraformaldehyde and quenched with 50 mmol/l NH_4_Cl for 5 min. After PBS washing, cells were permeabilised with 50 μg/ml digitonin for 10 min and blocked with 0.1% (wt/vol.) BSA for 30 min. The cells were incubated with the first antibodies and then washed and labelled with fluorescent secondary antibodies. The stained cells mounted in Mowiol (Sigma Aldrich, Saint Quentin Fallavier, France) were examined under a Zeiss LSM 510 confocal microscope (Zeiss, Marly le Roy, France) equipped with 63W oil objective and fluorescence was quantified using Image J software v1.56.

### Glutathione assay

Reduced glutathione (GSH) was measured using a commercially available kit from BioVision (Clinisciences, Montrouge, France) and normalised to the protein concentration measured using the Bradford assay.

### Statistics

Experimenters were not blind to group assignment. Data were analysed using Graphpad Prism (Graphpad Software, La Jolla, CA, USA) and presented as means ± SEM. Results were compared by one-way or two-way ANOVA followed when appropriate by post hoc Fisher PLSD tests. Simple comparisons were performed with Student’s *t* test using Welch’s correction for variance in homogeneity whenever needed. Differences were considered significant at the *p* < 0.05 level.

## Results

### 4-HHE accumulates in plasma from individuals with type 2 diabetes

**Fig. 1:**
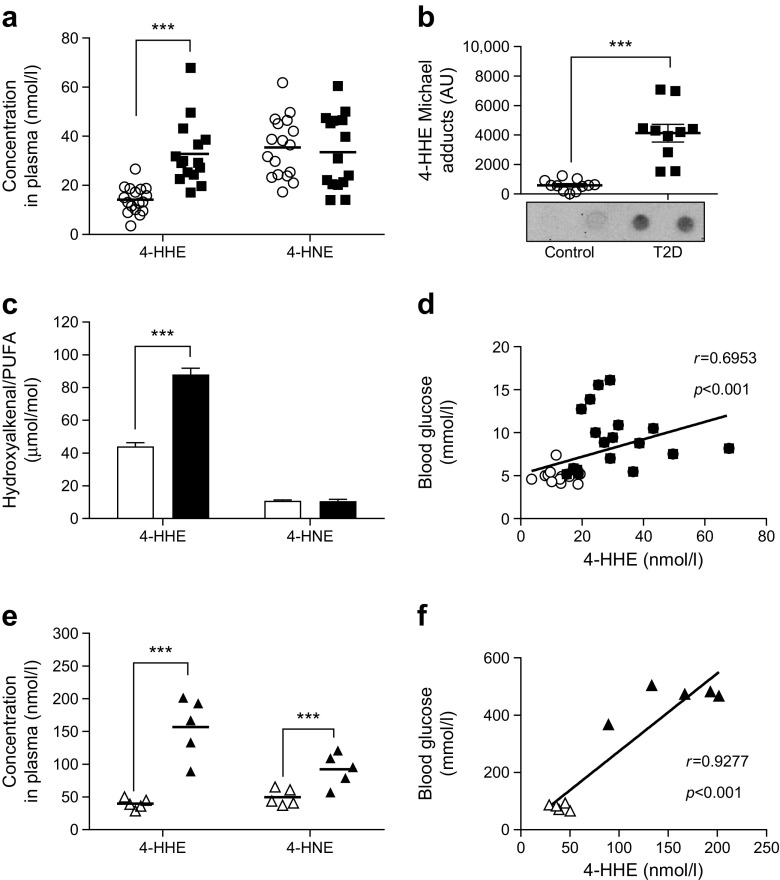
Plasma levels of 4-HHE are increased in type 2 diabetes. (**a**) Concentration of 4-HHE and 4-HNE in plasma from individuals with type 2 diabetes or from healthy volunteers, measured using GC–MS. (**b**) 4-HHE adducts on plasma proteins, measured by dot blot using anti-4-HHE Michael adduct antibody. AU, arbitrary units. (**c**) Normalisation of 4-HHE and 4-HNE to the concentration of *n*-6 PUFA and *n*-3 PUFA, respectively, measured by GC. (**d**) Correlation between plasma 4-HHE levels and blood glucose levels. (**e**) Concentration of 4-HHE and 4-HNE in plasma from obese ZDF rats (*n* = 5) or lean ZDF rats (*n* = 5), measured by GC–MS. (**f**) Blood glucose levels in the lean and obese ZDF rats measured using a glucometer. Data are means or means±SEM. ***p < 0.001 (two-way ANOVA). White circles, healthy volunteers (control); black squares, individuals with type 2 diabetes (T2D); white triangles, lean ZDF rats; black triangles, obese ZDF rats

The concentration of free 4-HHE, measured using GC–MS, was significantly increased in plasma from individuals with type 2 diabetes compared with plasma from healthy volunteers (33 vs 14 nmol/l, *p* < 0.001) (Fig. 1a). Diabetic individuals also exhibited a significant increase (sevenfold) in 4-HHE Michael adducts on plasma proteins (Fig. 1b). In contrast, 4-HNE levels did not differ between diabetic and healthy individuals; neither did the plasma concentrations of *n*-3 and *n*-6 fatty acids (data not shown). Normalisation of 4-HHE and 4-HNE to the concentration of their respective *n*-3 and *n*-6 precursors showed that one out of 20,000 *n*-3 PUFA were oxidised in the form of 4-HHE, while only one out of 100,000 *n*-6 PUFA were oxidised in the form of 4-HNE (Fig. 1c). Significant correlations with 4-HHE plasma concentrations were found for blood glucose levels (Fig. 1d) and for BMI, HbA_1c_, triacylglycerol and HDL-cholesterol (Table 3).

**Table 3:** Univariate correlations with 4-HHE plasma concentrations in participants

Variable	*r*	*p* value
Age, years	0.420	0.017
BMI, kg/m^2^	0.692	<0.001
SBP, mmHg	0.014	0.942
DBP, mmHg	−0.300	0.113
Fasting glucose, mmol/l	0.695	<0.001
HbA_1c_ (mmol/mol)	0.750	<0.001
Triacylglycerol, mmol/l	0.588	0.003
Total cholesterol, mmol/l	−0.302	0.184
HDL-cholesterol, mmol/l	−0.549	0.007
4-HNE, nmol/l	0.267	0.139
Total *n*-3 PUFA, mmol/l	0.643	<0.001
4-HHE Michael adducts, AU	0.805	<0.001

AU, arbitrary units; DBP, diastolic BP; SBP, systolic BP

### 4-HHE and 4-HNE in plasma from ZDF rats

A plasma concentration of 40 nmol/l of free 4-HHE was measured in lean ZDF rats and a fourfold increase (*p* < 0.001) was observed in obese ZDF rats (Fig. 1e). The plasma concentration of 4-HNE was increased by 80% in ZDF rats (*p* < 0.001). As in humans, a significant correlation was found between the concentration of 4-HHE and blood glucose levels (Fig. 1f).

### 4-HHE infusion induces insulin resistance in rats

**Fig. 2:**
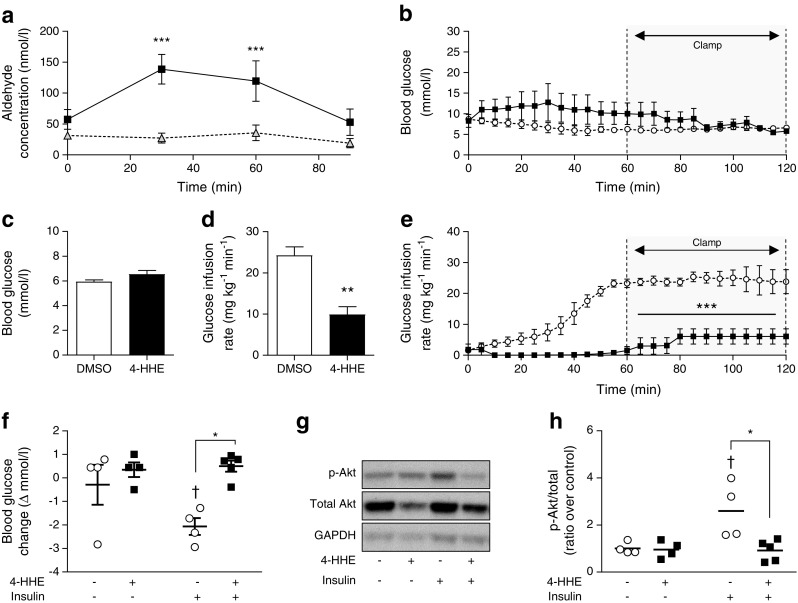
4-HHE infusion induces insulin resistance in rats. Euglycaemic–hyperinsulinaemic clamps were performed in anaesthetised Wistar rats infused with 4-HHE (10 mg/kg i.v.) or vehicle (DMSO) as described in Methods. (**a**) 4-HHE (black squares) and 4-HNE (grey triangles) concentration in 4-HHE-infused rats, measured in plasma over the 2 h of the clamp experiment. Results are means±SEM, *n* = 4. ***p < 0.001 vs time zero. (**b**) Blood glucose was monitored every 5 min using a glucometer and glucose infusion rate was adjusted accordingly. (**c**, **d**) Mean blood glucose (**c**) and glucose infusion rate (**d**) during the second hour of clamp. (**e**) In a separate experiment, rats were injected with 4-HHE (*n* = 5) or DMSO (*n* = 4) and after 2 h stimulated with insulin for 20 min; during euglycaemic–hyperinsulinaemic clamp, glucose infusion rate was adjusted accordingly over 2 h. (**f**) Blood glucose levels were measured with a glucometer. (**g**, **h**) Representative western blot (**g**) and quantification (**h**) of insulin-induced phosphorylation of Akt following 4-HHE and insulin injections. Data are means±SEM, *n* = 4. *p < 0.05, **p < 0.01 and ***p < 0.001 for indicated comparisons or vs DMSO; ^†^*p* < 0.05 vs no insulin (**d**–**f**, **h**). Black squares and bars, rats infused with 4-HHE; white circles and bars, rats infused with DMSO control

To demonstrate that 4-HHE plays a causal role in insulin resistance, insulin sensitivity was measured using euglycaemic–hyperinsulinaemic clamps in Wistar rats given an intravenous bolus of 4-HHE (10 mg/kg). The injection induced a transient increase in plasma concentration of 4-HHE, which reached 140 nmol/l 30 min after injection, while plasma 4-HNE concentration remained unaffected (Fig. 2a). The clamp was applied at a blood glucose level of approximately 6 mmol/l (Fig. 2b,c). The glucose infusion rate necessary to maintain euglycaemia was significantly lower in the 4-HHE infused rats than in the DMSO-infused rats (Fig. 2d,e), indicating whole-body insulin resistance. In another set of experiments, rats were infused with 4-HHE for 2 h and then received a single injection of insulin for 20 min. Under these conditions, the infusion of 4-HHE prevented the decreased in blood glucose level induced by insulin (Fig. 2f) and abolished insulin-induced phosphorylation of Akt in skeletal muscle (Fig. 2g,h).

### Impaired insulin-stimulated glucose uptake in response to 4-HHE

**Fig. 3:**
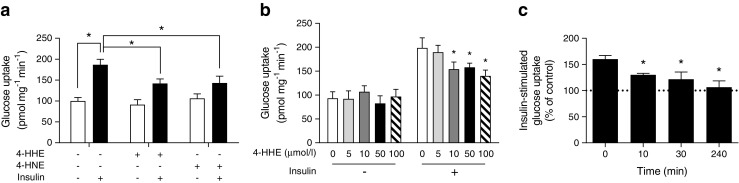
4-HHE impairs insulin-induced glucose uptake. Rat L6 muscle cells were treated with 4-HHE or 4-HNE and then stimulated with 100 nmol/l insulin for 20 min. 2-Deoxy-d-[^3^H]glucose uptake was measured as described in the Methods. (**a**) Glucose uptake in L6 muscle cells treated for 60 min with 50 μmol/l 4-HHE or 4-HNE. Data are means±SEM, *n* = 5. *p < 0.05 for indicated comparisons (two-way ANOVA for insulin vs aldehydes). (**b**) Glucose uptake in L6 cells treated for 30 min with increasing concentrations of 4-HHE. Data are means±SEM, *n* = 5. *p < 0.05 vs 0 μmol/l 4-HHE + 100 nmol/l insulin (two-way ANOVA). (**c**) Time course of insulin-stimulated glucose uptake in L6 cells in response to 10 μmol/l 4-HHE. Data are means±SEM, *n* = 5. *p < 0.05 vs 0 min (one-way ANOVA)

In lean animals, 80–90% of infused glucose is taken up by skeletal muscle [28] so the clamp technique primarily reflects skeletal muscle insulin sensitivity. We therefore went on to characterise 4-HHE-induced insulin resistance in skeletal muscle cells. In rat L6 cells, 4-HHE completely blunted insulin-induced 2-deoxy-d-[^3^H]glucose uptake (Fig. 3a), with maximal inhibition observed starting at 10 μmol/l (Fig. 3b), lower than the concentration of 4-HNE previously reported to impair insulin response in adipocytes and muscle cells [14, 15]. Insulin-induced glucose uptake was similarly blunted in L6 cells exposed to 4-HNE, but with a maximal inhibition obtained at a concentration of 50 μmol/l*.* Significant inhibition of insulin-induced glucose uptake by 4-HHE was observed from 10 min until 4 h of treatment (Fig. 3c). In cells treated for 30 min with up to 100 μmol/l 4-HHE, MTT reduction, lactate dehydrogenase (LDH) release and caspase-3 activity remained unaffected, demonstrating no deleterious effects on cell viability at times and concentrations used (Table 4).

**Table 4:** Effect of 4-HHE and 4-HNE on rat L6 muscle cell viability

Aldehyde concentration	Cell viability (% of control)	LDH activity (% of total)	Caspase 3 activity (% of control)
4-HHE			
10 μmol/l	108 ± 2	0.9 ± 0.2	116 ± 2
25 μmol/l	101 ± 3	1.5 ± 0.8	117 ± 6
50 μmol/l	107 ± 4	0.9 ± 0.2	116 ± 12
75 μmol/l	120 ± 5	1.8 ± 0.7	119 ± 14
100 μmol/l	125 ± 1	1.1 ± 0.3	121 ± 12
4-HNE			
10 μmol/l	105 ± 5	2.1 ± 0.4	115 ± 16
25 μmol/l	105 ± 3	2.6 ± 0.6	122 ± 15
50 μmol/l	108 ± 5	2.4 ± 0.9	116 ± 13
75 μmol/l	109 ± 3	2.4 ± 0.9	119 ± 19
100 μmol/l	106 ± 2	4.1 ± 1.3	140 ± 20

Data are means±SEM from four independent experiments

L6 muscle cells were treated for 30 min with increasing doses of 4-HHE or 4-HNE in serum-free conditions. Cell viability was measured by MTT assay and expressed as a percentage of untreated control. LDH activity in the supernatant fraction was expressed as a percentage of the maximal level of LDH activity determined after total cell lysis. Caspase-3 activity was expressed as a percentage of the specific activity present in vehicle-treated cells

No significant effects were found

### Impaired insulin signalling induced by 4-HHE in rat L6 muscle cells

**Fig. 4:**
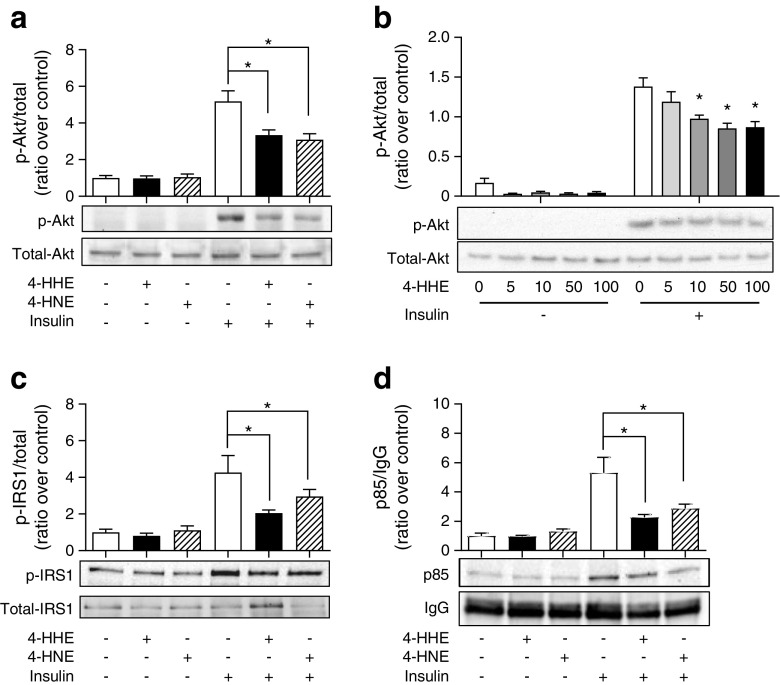
4-HHE impairs insulin signalling. Rat L6 cells were treated for 30 min with 50 μmol/l 4-HHE or 4-HNE and then stimulated with 100 nmol/l insulin for 20 min. Protein extracts were analysed by western blotting. (**a**) Phosphorylation of Akt. (**b**) Phosphorylation of Akt in response to increasing concentrations of 4-HHE. (**c**) Tyrosine phosphorylation of IRS1. (**d**) p85 co-immunoprecipitated with IRS1. Data are expressed as means±SEM, *n* = 4. *p < 0.05 for indicated comparison or vs insulin-stimulated cells untreated with 4-HHE (two-way ANOVA in **a**, **c** and **d**; one-way ANOVA in **b**)

The insulin signalling cascade leading to glucose uptake can be affected at various stages: GLUT4 translocation; protein kinase B (PKB)/Akt and/or upstream at the insulin receptor and IRS. In response to 4-HHE or 4-HNE, insulin-induced phosphorylation of PKB/Akt was reduced by 50% (Fig. 4a). 4-HHE was effective at inhibiting insulin-induced Akt phosphorylation at concentrations ranging from 10 to 100 μmol/l (Fig. 4b). Similarly, exposure to 4-HHE significantly impaired insulin-induced IRS1 phosphorylation (Fig. 4c), in parallel with a marked reduction in the level of p85 protein co-immunoprecipitated with IRS1 (Fig. 4d).

### 4-HHE generates protein adducts in rat L6 muscle cells

**Fig. 5:**
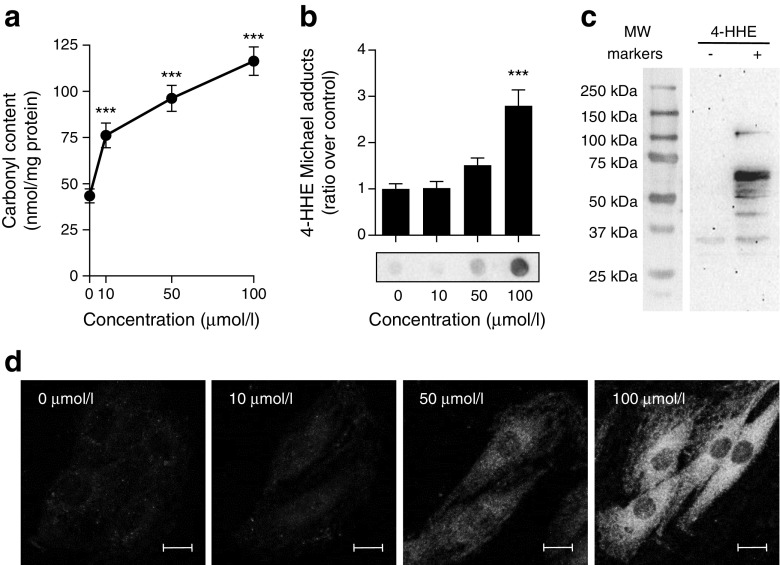
4-HHE-induced carbonylation in muscle cells. Rat L6 cells were exposed to 4-HHE for 30 min. (**a**) Carbonylation of proteins, estimated using DNPH assay. Data are means±SEM, *n* = 3. (**b**, **c**) Adduct formation on proteins was determined by dot blot (**b**) and western blot (**c**) using antibodies against 4-HHE Michael adducts. Data are means±SEM, *n* = 6. Representative blots are shown. (**d**) Immunofluorescence was performed with confocal microscopy using antibodies to 4-HHE adducts. Representative images from at least five different fields are shown. Scale bar, 10 μm. ***p < 0.001 vs no 4-HHE (one-way ANOVA). MW, molecular weight

Many deleterious effects of aldehydes have been attributed to their ability to form covalent adducts on biomolecules [5]. Indeed, incubation of L6 muscle cells for 30 min with 4-HHE resulted in a dose-dependent increase in carbonyl content (Fig. 5a) and increased formation of 4-HHE Michael adducts on proteins (Fig. 5b). Western blot analysis revealed the presence of 4-HHE adducts on many proteins from 30 to 100 kDa (Fig. 5c) and confocal microscopy showed that the formation of adducts was diffuse in the cell cytoplasm but was excluded from the nuclei (Fig. 5d) without any specific co-localisation with actin cytoskeleton or the Golgi apparatus (data not shown). These data demonstrate that 10 μmol/l 4-HHE is sufficient to induce carbonylation of cytoplasmic proteins in muscle cells.

### Elevation of glutathione pools reverses 4-HHE-impaired glucose uptake

**Fig. 6:**
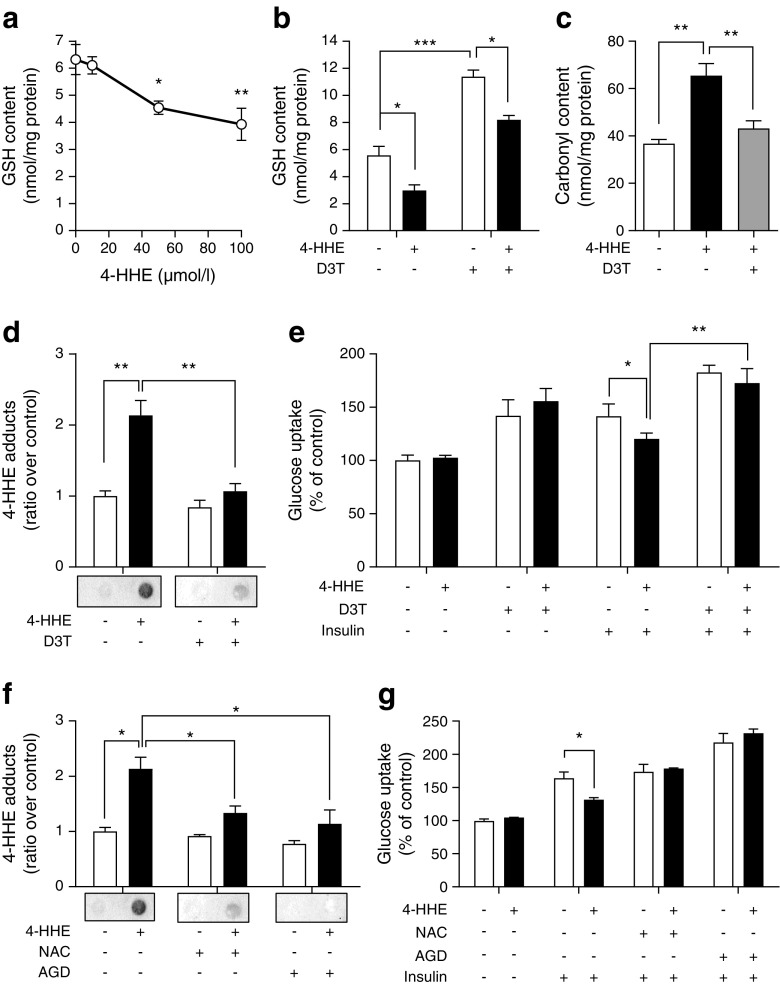
Increasing glutathione levels reverses 4-HHE-induced adduct formation and impaired glucose uptake in muscle cells. (**a**) GSH was measured in rat L6 muscle cells using a commercially available kit. Data are means±SEM, *n* = 3. (**b**) L6 cells were pre-incubated with 100 μmol/l of D3T for 24 h and then treated with 50 μmol/l 4-HHE for 30 min. GSH was measured as described in Methods. Data are means±SEM, *n* = 3. (**c**) After D3T pre-incubation, L6 cells were exposed to 50 μmol/l 4-HHE for 30 min. Carbonylation of proteins was assayed using the DNPH assay. Data are means±SEM, *n* = 4. (**d**) Michael adducts on proteins were detected with a dot blot using antibodies to 4-HHE adducts. Data are means±SEM, *n* = 3. (**e**) After D3T pre-incubation and 4-HHE exposure, insulin-stimulated glucose uptake was measured using 2-deoxy-d-[^3^H]glucose. Data are means±SEM, *n* = 4. (**f**, **g**) L6 cells were pre-incubated with 1 mmol/l *N*-acetyl-cysteine (NAC) or 5 mmol/l aminoguanidine (AGD) for 24 h and then treated with 50 μmol/l 4-HHE for 30 min. Adducts on proteins were detected by dot blot (**f**) and glucose uptake was measured using 2-deoxy-d-[^3^H]glucose (**g**). Data are means±SEM, *n* = 4. *p < 0.05, **p < 0.01, ***p < 0.001 for indicated comparisons or vs no 4-HHE (one-way ANOVA in **a**, **c**; two-way ANOVA in **b**, **d**–**g**)

Reduced glutathione (GSH) is one of the major aldehyde detoxification mechanisms [29]. Treatment with increasing concentrations of 4-HHE indeed resulted in a sharp dose-dependent decrease in GSH content in rat L6 muscle cells (Fig. 6a). This decrease was reversed by pre-treatment with D3T (100 μmol/l, 24 h), which doubled the intracellular GSH content and counteracted the decrease induced by 4-HHE (Fig. 6b). D3T also reversed the 4-HHE-induced increase in total carbonyl content (Fig. 6c) and protein adducts in the cells (Fig. 6d). Pre-treatment with D3T increased the basal glucose uptake and effectively prevented 4-HHE-induced insulin resistance (Fig. 6e). Similarly, pre-treatment of L6 muscle cells with *N*-acetyl-cysteine (NAC) or the scavenger of lipid peroxidation by-products aminoguanidine (AGD) also attenuated Michael adduct formation (Fig. 6f) and prevented the impaired insulin-induced glucose uptake (Fig. 6g) induced by 4-HHE. Altogether, these data confirm that adduct formation is a major player in 4-HHE-induced insulin resistance.

## Discussion

Diabetes is associated with increased oxidative stress in metabolic tissues and excessive production of reactive oxygen species negatively affects insulin responses [2]. Oxidative stress can impair cell function through direct attack by reactive oxygen species and via the production of reactive oxidation by-products. Here, we demonstrate that one of the by-products of *n*-3 PUFA peroxidation, 4-HHE, is increased in plasma during type 2 diabetes, induces insulin resistance in vivo and impairs glucose uptake and signalling in skeletal muscle cells in vitro.

Unlike radical oxygen species, lipid peroxidation by-products are long-lived and may spread from their site of production to exert their effects throughout the whole organism. Previous reports show that the plasma concentration of free 4-HNE ranges from 70–600 nmol/l at baseline to up to 2–10 μmol/l under pathological conditions [11, 30, 31]. In our study, the 4-HNE plasma concentration in healthy volunteers was 50 nmol/l and was surprisingly unchanged in individuals with type 2 diabetes. On the contrary, 4-HHE concentration was doubled in individuals with type 2 diabetes compared with healthy individuals. The measurement of free 4-HHE or 4-HNE by GC does not take into account the amount of aldehydes bound to biomolecules. There are no commercially available ELISA kits for 4-HHE adducts, so we used dot blots to demonstrate that plasma proteins from individuals with type 2 diabetes exhibit higher levels of 4-HHE Michael adducts (sevenfold increase). The accumulation of protein adducts (carbonyls) has been reported in individuals with type 2 diabetes; urinary levels of acrolein adducts are increased and significantly correlated with control of blood glucose [32]. A previous study has reported a fivefold increase in 4-HHE Michael adducts on phospholipids during diabetic retinopathy [33]. Our study therefore reinforces the evidence for increased production of aldehyde by-products and their adducted targets during diabetes.

Daily supplementation with 800–1600 mg docosahexaenoic acid (DHA) in healthy volunteers significantly increased plasma free 4-HHE (from 9 to 93 nmol/l), likely resulting from increased lipid peroxidation [34]. Interestingly, a DHA supplement that did not significantly increase plasma 4-HHE (400 mg DHA/day) had beneficial effects on platelet function and induced antioxidant effects [35], more recently confirmed in individuals with type 2 diabetes [36]. In human volunteers who take *n*-3 PUFA supplements, the balance between the beneficial effects of DHA supplementation and the potential deleterious effects of its by-products is therefore difficult to assess and might depend on the concentration of DHA as well as the oxidative environment.

Data from animal models and cell culture suggest that oxidative stress plays a causative role in the development of type 2 diabetes. Reactive oxygen species and their by-products can have a negative impact on insulin sensitivity [2] and we demonstrate here that 10 μmol/l 4-HHE is sufficient to impair insulin-induced glucose uptake. In rat L6 muscle cells, impaired glucose uptake likely results from an alteration of insulin-induced PKB/Akt phosphorylation on serine 473 as well as IRS1 tyrosine phosphorylation and p85 docking. Insulin-induced IRS1 phosphorylation can be counteracted by serine phosphorylation conducted for instance by mitogen-activated protein kinase [37, 38]. However, extracellular signal-regulated kinase and c-Jun N-terminal kinases were only mildly activated by 4-HHE in L6 cells (data not shown), suggesting that these kinases do not play a major role in the impairment of insulin signalling in this context. On the contrary, we detected a significant accumulation of Michael adducts in L6 muscle cells exposed to 4-HHE, suggesting that the major effects of 4-HHE are due to protein carbonylation. Detoxification of aldehydes in cells is fulfilled by several enzymes and antioxidant systems. Compared with 4-HNE, 4-HHE is a poor substrate for aldehyde dehydrogenase 5A [39] but is metabolised to GSH adducts more efficiently [40], suggesting that GSH metabolism may be the major mechanism for detoxification of 4-HHE. In our study, prevention of carbonylation either by increasing glutathione pools with D3T or by using the by-product scavenger aminoguanidine reversed 4-HHE-induced insulin resistance. This favours the notion that 4-HHE impairs insulin signalling through the formation of covalent adducts on key proteins and that GSH is a major means by which to prevent its deleterious effects. In 3T3 adipocytes, 4-HNE directly binds IRS1 and promotes its degradation [14]; however, we did not observe any change in the total amount of PKB/Akt and IRS1 proteins in response to 4-HHE. It is therefore likely that 4-HHE exerts its noxious effects through adduction of proteins but not through the specific degradation of proteins of the insulin signalling pathway.

In conclusion, we demonstrate that plasma levels of 4-HHE are significantly increased in type 2 diabetes and that 4-HHE can significantly impede insulin action in vitro and in vivo. We also report that increasing the GSH pool is an efficient way to prevent 4-HHE-induced carbonylation of cellular proteins and impairment of insulin signalling. These data support the idea that lipid peroxidation by-products, especially 4-HHE, can significantly contribute to the development of type 2 diabetes and could represent a therapeutic target to taper insulin resistance.

## Data Availability

The data generated during and/or analysed during the current study are available from the corresponding author on reasonable request.

## Abbreviations

DHA: Docosahexaenoic acid
D3T: 1,2-Dithiole-3-thione
DNPH: 2,4-Dinitrophenylhydrazine
EIA: Enzyme immunoassay
GSH: Reduced glutathione
4-HHE: 4-Hydroxy-2-hexenal
4-HNE: 4-Hydroxy-2-nonenal
LDH: Lactate dehydrogenase
MTT: 3-(4,5-Dimethylthiazol-2-yl)-2,5-diphenyltetrazolium bromide
PKB: Protein kinase B
PUFA: Polyunsaturated fatty acids
ZDF: Zucker diabetic fatty
